# Facilitators and barriers to the regulation of medical cannabis: a scoping review of the peer-reviewed literature

**DOI:** 10.1186/s12954-021-00547-8

**Published:** 2021-10-14

**Authors:** Mohammad Ali Ruheel, Zoya Gomes, Sana Usman, Pargol Homayouni, Jeremy Y. Ng

**Affiliations:** Department of Health Research Methods, Evidence, and Impact, Faculty of Health Sciences, McMaster University, Michael G. DeGroote Centre for Learning and Discovery, Room 2112, 1280 Main Street West, Hamilton, ON L8S 4K1 Canada

**Keywords:** Medical cannabis, Medical marijuana, Scoping review, Regulation, Policy

## Abstract

**Background:**

In recent decades, several political, legislative, judicial, consumer, and commercial processes around the world have advanced legalization efforts for the use of medical cannabis (MC). As the use of MC evolves through legislative reform, with an increase in public acceptance and therapeutic potential, a need exists to further investigate the facilitators and barriers to MC regulation.

**Methods:**

A scoping review was conducted to identify the facilitators and barriers associated with the implementation of MC regulations. MEDLINE, EMBASE, AMED and PsycINFO databases were systematically searched; no restrictions were placed on geographic location/jurisdiction. Eligible articles included those that evaluated the MC regulatory framework of one or more countries.

**Results:**

Twenty-two articles were deemed eligible and included in this review. Themes identified include: (1) effects of conflicts, mindset, and ideology of state population, (2) the use of comparisons to analyze MC regulation, and (3) the need for more knowledge, advice, and empirical/clinical evidence to inform future MC policies.

**Conclusion:**

Policymakers should be aware of facilitators to the MC regulation implementation process, such as the influence of state and federal congruence, increased transparency, and the incorporation of stakeholder concerns, in order to effectively respond to a growing societal acceptance of MC and its use among patients. Given a comprehensive understanding of these influential factors, policymakers may be better equipped to meet the consumer and commercial demands of a rapidly evolving MC regulatory environment.

## Background

The use of medical cannabis (MC) dates back thousands of years, first appearing in a medical text composed by Emperor Shen-Nung around 2800 BCE documenting its potential in treating various conditions [1]. By the nineteenth century, an increase in the recreational use of cannabis prompted the emergence of various prohibition laws and policies due to controversies surrounding its legal, ethical, and societal implications [2]. Opponents of MC legalization in the twentieth and twenty-first centuries cited its addictive properties, associations with criminal behaviours, and a lack of medical evidence as drivers for its sustained illegality [3]. In the 1950s and 1960s, however, the budding popularization of recreational cannabis also propelled scientific investigation into its therapeutic capacities [1]. In recent decades, several political, legislative, judicial, consumer, and commercial processes around the world have advanced legalization efforts for the use of MC [1, 4]. In 1999, Canada became one of the first nations to initiate a centralized MC program and provide MC prescriptions to patients [1, 5]. Similarly, in 1996, California became the first US state to legalize MC and by 2016, this transition had been consolidated across the majority of the US [4]. There is also a continuous pressure for the implementation of MC laws beyond North American jurisdictions. In 2019, Thailand amended its original law, the *Narcotic Act B.E. 2522*, shifting from the strict prohibition of cannabis to legalizing its medical use under certain conditions [6]. Further, in 2013, Uruguay became the first country to legalize cannabis through government public health efforts, while in countries such as Argentina, Brazil and Mexico, legislative reforms have not adapted to achieve the same extent of regulation [7]. As of today, MC programs have been established in various countries around the world, including but not limited to Canada, Colombia, Chile, Germany, Israel, Italy, Jamaica, the Netherlands, Switzerland, Thailand, the United Kingdom (UK), Uruguay, and more than 30 US states [6].

While some researchers continue to express concern over cannabis use including possible adverse outcomes, potential for dependence, and associated national stigma, the usage of MC continues to rise [2]. It is estimated that over 3.5 million Americans used MC legally in 2019 and Canadian MC prescriptions tripled from 30 537 in 2015 to 100 000 in 2016 [5]. Additionally, the Netherlands has seen a dramatic increase in patients using MC since its legalization in 2000, with over 50 000 patients now being prescribed MC [7]. Other countries including Greece, Poland, and Slovenia are following closely behind and have adopted various MC regulation schemes [7]. Furthermore, in 2017, due to the growing popularity of MC, New Zealand introduced the *Misuse of Drugs Amendment Bill* with the objective of making MC accessible to citizens without criminal liability [7].

Although a reasonable degree of clinical ambiguity surrounding the benefits and challenges of MC continues to exist, ongoing research has shown promise for the use of MC in treating various medical conditions [5]. Several randomized controlled trials have assessed the effects of MC on fibromyalgia, epilepsy, traumatic brain injury, neurological disorders, and a considerable number of other conditions and symptoms [5]. Current literature outlines that there exists substantial evidence for the efficacy of MC in treating conditions including chronic pain and multiple sclerosis-related spasticity, with conclusive or limited evidence for symptoms such as cancer-related nausea [5]. As MC prohibition continues to be lifted through legislative action, certain populations have demonstrated an increasing acceptance of MC usage. In 2016, a Quinnipiac University poll found that 81 percent of American respondents were in favor of MC legalization [2]. In addition, citizens of Israel and Norway have shown an increase in public support for MC legalization due to increasing beliefs about the potential medical effects of cannabis [8].

There has been significant controversy surrounding the implementation of centralized national medical cannabis programs in countries including Canada, the US, and the Netherlands, with criticism from judicial courts, medical establishments, law enforcement and consumers themselves [1]. Further regulation of MC will depend on the expansion of clinical research programs, government cooperation, and community-based strategies in order to promote safe and equitable access [1]. Without a clear understanding of its regulatory history, conflicting evidence and unrelenting media attention can present MC policy formulation and implementation as an incredibly difficult task [9].

To our knowledge, there is no current literature that summarizes the facilitators and barriers to MC regulation, which takes into account no restriction on country or jurisdiction. Given that there is a growing body of evidence to suggest that MC use is evolving through legislative actions, with an increase in public acceptance and therapeutic potential, there is a need for further investigation into the factors that affect MC policy formulation. As such, the objective of this scoping review is to identify and summarize the facilitators and barriers to MC regulation throughout different parts of the world.

## Methods

### Approach

A scoping review was conducted to identify the facilitators and barriers to the regulation of MC; the scoping review was informed by Arksey and O’Malley’s [10] framework, and further supplemented by Levac et al. [11] and Daudt et al. [12]. The methodology consists of five-stages, as follows: (1) identifying the research question; (2) identifying relevant studies; (3) selecting the studies; (4) charting the data, and (5) collating, summarizing, and reporting the results [10, 11]. We chose this method as it provided an outline for searching and evaluating current literature on the present topic, determining eligibility criteria, and summarizing eligible article content to identify themes and highlight knowledge gaps. Our registered study protocol on Open Science Framework can be found at: https://doi.org/10.17605/OSF.IO/6HGRX.

### Step 1: identifying the research question

The research question for this scoping review was as follows: “What facilitators and barriers to the regulation of MC can be identified by studies evaluating policies and regulatory frameworks across different countries?”. For this study, we defined a “facilitator” as any factor(s) that allow(s) or promote(s) the implementation of MC regulation. An example of a facilitator to MC regulation can include the congruence of federal and state laws regarding MC usage. On the other hand, we defined a “barrier” as any factor(s) that prevent(s) or hinder(s) the implementation of MC regulation. Some general examples of barriers to MC regulation can include MC stigma, negative public mindset, or a lack of knowledge surrounding MC usage and benefits.

### Step 2: identifying relevant studies

Following a preliminary scan of the literature, we conducted systematic searches on June 01, 2020 from database inception until May 29, 2020 on MEDLINE, EMBASE, AMED, and PsycINFO. The search strategy included indexed headings and terms used in the literature to refer to MC regulation. A sample search strategy can be found in Table 1. We elected not to search the grey literature, as we specifically wanted to identify facilitators and barriers identified by policy evaluations, which are academic studies likely to be published only in the peer-reviewed literature.

**Table 1:**
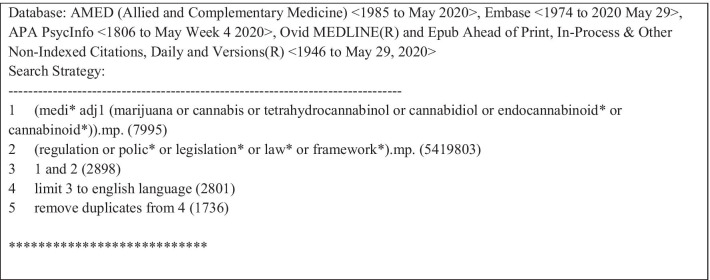
OVID search strategy executed June 01, 2020

### Step 3: selecting the studies

Our preliminary scans of the academic literature allowed us to identify some eligible articles. We included all primary research articles, research protocols, and review articles that evaluated MC regulations in one or more countries globally. In this case, we define a “review” as an article that has examined barriers and/or facilitators to the implementation of MC policy formulation or regulation, and also included those that were not systematic or scoping reviews. The reference lists of ineligible review articles were read to source additional articles that could potentially meet our eligibility criteria. Exclusion criteria included content outside of MC or articles which solely focused on the recreational use of cannabis. Articles in the form of commentaries, editorials, letters to the editor, conference abstracts, and opinion pieces were also ineligible. Further, our eligibility criteria restricted our search to articles published solely in the English language and those publicly accessible or retrievable through a library system. SU and PH independently pilot-screened all retrieved articles to first determine eligibility based on title and abstract. A second round of screening was then conducted to determine eligible articles based on their full text. Each round of independent screening was followed by a discussion between JYN, SU, and PH to reach a consensus on which articles were considered eligible and to determine any inconsistencies or questions regarding the inclusion criteria. Discrepancies were resolved by discussion.

### Step 4: charting the data

We developed a data extraction form that allowed us to evaluate each article and to identify any relevant information. The following data from each eligible article was summarized and extracted: author(s); year of publication; study design; country/countries discussed; the objective of the study; participants/level of policy-making; types of evidence from which the evaluation of the MC regulation was made; summary of methods; facilitators to the implementation of MC regulations; and barriers to the implementation of MC regulations. Following data extraction being conducted independently and in duplicate, JYN, SU, and PH met to discuss and resolve discrepancies. The aim of the data extraction was to collect and analyze information from included studies required to identify relevant themes and subthemes.

### Step 5: collating, summarizing, and reporting the results

Charted data was summarized in the format of tables, and the descriptive data was analyzed using content analysis. MAR, ZG, and JYN reviewed the descriptive data and resolved any discrepancies through discussion. JYN identified codes relative to the findings, and all authors subsequently organized the findings into themes.

## Results

**Fig. 1:**
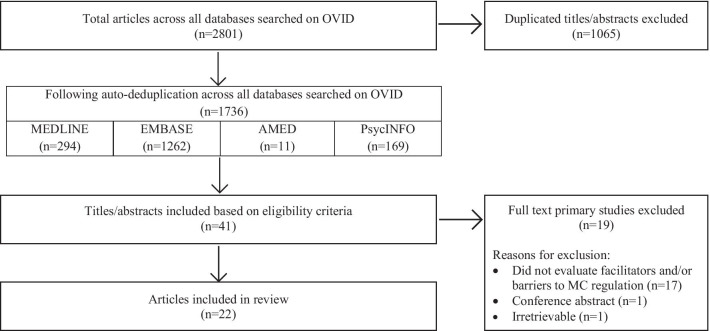
PRISMA Diagram. MEDLINE, EMBASE, AMED, and PsycINFO search results are recorded following deduplication using the “deduplicate” function on OVID. Records after duplicates removed are reported based on a subsequent manual deduplication of all records

Searches generated a total of 2801 results, 1065 of which were duplicates; following title/abstract screening, 1695 items were excluded, leaving 41 full-texts to be assessed. After full-text screening, 19 items failed to meet the eligibility criteria because they did not evaluate the facilitators and/or barriers to MC regulation (*n* = 17), were a conference abstract (*n* = 1), or were irretrievable (*n* = 1), resulting in a total of 22 eligible articles that were included in this scoping review. A PRISMA diagram detailing this process is shown in Fig. 1.

### Eligible article characteristics

Eligible articles were published between  2002 and 2019 inclusive, and originated from the US (*n* = 16), Canada (*n* = 2), Israel (*n* = 2), Japan (*n* = 1), and the UK (*n* = 1). The countries discussed included the US (*n* = 16), Canada (*n* = 3), Israel (*n* = 2), Germany (*n* = 1), Jamaica (*n* = 1), Japan (*n* = 1), UK (*n* = 1), and Uruguay (*n* = 1). The 22 eligible articles had varying study designs and some studies employed multiple designs; while the majority of articles were policy analyses (*n* = 14), there were also interviews (*n* = 5), literature reviews (*n* = 4), surveys (*n* = 4), case studies (*n* = 3), and a comparative analysis (*n* = 1). Specifically, the comparative analysis examined laws, regulations, and discussions with regulators and functionaries of each jurisdiction. Many of the policy analyses touched upon medical marijuana laws (MMLs) specific to each country on a federal or state level while drawing upon key information from interviews with stakeholders in the MC policy field. Additionally, interviews and/or surveys were common methods used to gather evidence to evaluate MC programs. The case studies analyzed regulatory acts, political structures, and archival advisory material. Further details associated with all eligible articles referenced in this review, including study objectives and study design can be found in Table 2. A summary of study methods, types of evidence, and facilitators and barriers to MC regulation across eligible articles can be found in Table 3.

**Table 2:** Eligible article characteristics (*n* = 22)

First author and year	Article title	Country of first author	Countries discussed	Study design	Study aim
Ablin et al. 2016 [27]	Medical use of cannabis products: Lessons to be learned from Israel and Canada	Israel	Canada, Germany and Israel	Policy analysis	To examine the different aspects of MC and regulatory frameworks surrounding MC, as well as, how these regulations can be applied in Germany
Bradford et al. 2017 [3]	Factors driving the diffusion of medical marijuana legalisation in the United States	United States	United States	Policy analysis	A review of how the adoption of medical marijuana laws spread across the US, along with a review of the factors affecting the policy diffusion
Campbell et al. 2015 [26]	The medical marijuana catch-22: How the federal monopoly on marijuana research unfairly handicaps the rescheduling movement	United States	United States	Policy analysis	To analyze MC policies in the US and the categorization of cannabis as a Schedule I drug
Choo et al. 2017 [18]	Clearing the haze: The complexities and challenges of research on state marijuana laws	United States	United States	Literature review	To provide an overview of marijuana laws in the US, in addition to a review of the status of policy research in the US while highlighting issues and the considerations brought to attention during the policy development process; to examine areas in the policy development process that require further research and development
Cohen et al. 2010 [28]	Medical marijuana 2010: It's time to fix the regulatory vacuum	United States	United States	Literature review	To conduct a literature review on the history of MC laws in the US, as well as, to provide an overview of the current MC laws
Davenport et al. 2016 [30]	The Dangerous Drugs Act Amendment in Jamaica: Reviewing goals, implementation, and challenges	United States	Jamaica and the United States	Policy analysis	To analyze how the Dangerous Drug Amendment Act influences populations across Jamaica, achieves its stated goals, and the various challenges faced by the implementation process of the regulatory framework
Flexon et al. 2019 [19]	The effect of cannabis laws on opioid use	United States	United States	Policy analysis	To investigate the effect of medicinal marijuana laws on opioid users, and whether individuals with chronic pain are opting to use medicinal marijuana instead of opioids due to the law changes
Grbic et al. 2017 [20]	Observations of the role of science in the United States medical cannabis state policies: Lessons learnt	United States	United States	Policy analysis	To identify the main challenges to the development of MC laws in the US
Hammer et al. 2015 [21]	Patients, police, and care providers: The cultural dimension of medical marijuana implementation	United States	United States	Policy analysis	To test the social construction theory and utilize the Institutional Analysis and Design framework to determine the role political culture has on MC regulation and implementation
Heddleston et al. 2013 [15]	A tale of three cities: Medical marijuana, activism, and local regulation in California	United States	United States	Case study	To examine the MC policies and regulations in three US cities, including Los Angeles, San Diego, and San Francisco using three different models
Kim et al. 2018 [13]	Policy innovation and diffusion through policy typologies: Examining the predictors of medical marijuana legalization in states	United States	United States	Policy analysis	To evaluate factors that contribute to the diffusion of medical marijuana laws; the type of policies that determines whether a medical marijuana policy is adopted or not; and, how the adoption of medical marijuana laws affects the legalization of medical marijuana in the state
Lamonica et al. 2016 [9]	Gaps in medical marijuana policy implementation: Real-time perspectives from marijuana dispensary entrepreneurs, health care professionals and medical marijuana patients	United States	United States	Policy analysis	To review the recent changes in marijuana policy in Massachusetts and examine the challenges for its implementation
Lucas et al. 2008 [1]	Regulating compassion: An overview of Canada's federal medical cannabis policy and practice	Canada	Canada	Policy analysis	To examine the origin and evolution of Health Canada's MMAD, Canadian Institute of Health Research Medical Marijuana Research Program and Prairie plant system, and the Canadian federal contract
Lucas et al. 2012 [22]	It can't hurt to ask; A patient-centered quality of service assessment of Health Canada's medical cannabis policy and program	Canada	Canada	Survey and Interviews	To gain knowledge about the needs of patients who have been federally authorized for MC, as well as, the challenges and experiences associated with Health Canada's Marihuana Medical Access Division
Miyaji et al. 2016 [23]	Tackling the pharmaceutical frontier: Regulation of cannabinoid-based medicines in postwar Japan	Japan	Japan	Literature review	To explore the history of the development of MC and discuss the control of cannabis and the status of research on MC in Japan
Pacula et al. 2002 [31]	State medical marijuana laws: Understanding the laws and their limitations	United States	United States	Policy analysis	To present original legal research on current MC laws and regulations in all 50 states in the US
Pacula et al. 2014 [14]	Words can be deceiving: A review of variation among legally effective medical marijuana laws in the United States	United States	United States	Literature review	To determine the characteristics of MMLs that have been established in 1990; specifically, the study examined factors associated with consumers/patients, the influence of the MMLs on access, and an overall examination of the variation amongst the laws
Pardo et al. 2014 [24]	Cannabis policy reforms in the Americas: A comparative analysis of Colorado, Washington, and Uruguay	United States	United States and Uruguay	Policy analysis	To explore policies on cannabis legalization in Uruguay and Colorado and Washington, and to provide policymakers with data that allowed for a deeper understanding of the policies and challenges associated with cannabis reform
Smith et al. 2013 [17]	Medical marijuana regulation in Michigan: Problems of legitimacy	United States	United States	Case study	To analyze the Michigan Medical Marijuana Act (MMMA) and examine the social issues that arose during the development process of the regulation
Taylor et al. 2016 [25]	Evidence-based policy? The re-medicalization of cannabis and the role of expert committees in the UK, 1972–1982	United Kingdom	United Kingdom	Case study	To examine the role of UK-based expert groups during the implementation process of MC policies from 1972 to 1982
Tilburg et al. 2019 [16]	Emerging public health law and policy issues concerning state medical cannabis programs	United States	United States	Policy analysis	To examine how restrictions on cannabis, imposed by federal laws, impede the development of regulatory frameworks for cannabis ultimately affect the patients and the public
Zarhin et al. 2018 [29]	Rhetorical and regulatory boundary-work: The case of medical cannabis policy-making in Israel	Israel	Israel	Policy analysis	To examine the MC policymaking process in Israel; the development process was then reviewed to determine the boundaries that dictated between the legal and illegal use of MC

**Table 3:** Summary of methods, types of evidence, facilitators and barriers

First author and year	Summary of description of methods	Types of evidence	Facilitators to the implementation of MC regulations	Barriers to the implementation of MC regulations
Ablin et al. 2016 [27]	The authors analyzed the medicinal cannabis regulatory frameworks in Canada and Israel. The analysis also included evaluations of the utilization, barriers, and unmet needs that exist in these countries. Finally, the authors examined the process of implementing these regulations in Germany.	The authors analysed the MMLs from Israel and Canada.	MC programs should collaborate with the medical community regarding the use of MC. Further; the medical community should be involved in the implementation and decision-making process. MC regulatory frameworks should be clear and address inconsistencies in MC policies.	Federal laws that act as a resistance to providing funding and creating opportunities for research on MC.
Bradford et al. 2017 [3]	The authors created an empirical model of policy adoption to determine factors that helped policy diffusion. This was done using an Event History Analysis. For the model, data on the status of states MMLs were obtained from several sources. Finally, the model allowed the authors to examine the policy demand and the role of policy diffusion forces and median voters on a state adopting a particular policy.	The data for this study was extracted from various sources including the Bureau of Labor Statistics, the Marijuana Policy Project, the Centers for Disease Control and Prevention, and the Office of National Drug Control Policy's Marijuana Resource Center. Moreover, the study also examined the National Conference of State Legislature, the United States Census Bureau, and the National Institute of Education Services.	States with neighbouring states that have implemented MMLs, states with liberal-minded citizens, and states that have a higher median household income are all more likely to implement MMLs.	The likelihood to adopt new regulations decreases as motivational effects decrease between those who wish to adopt and those who do not.
Campbell et al. 2015 [26]	The authors analyzed Americans for Safe Access, Craker's Petition, the Controlled Substances Act, and the role of research in the context of the MMLs.	The authors reviewed and analyzed the following data: American's for Safe Access, Craker's Petition, and the Controlled Substances Act.	Changing the Controlled Substance Act (CSA) so that the federal MC regulations are consistent with the classification of the drug with state-level regulations.	The inconsistency between the federal classification of MC as a Schedule 1 drug and state laws prevents opportunities for clinical and empirical research on MC.
Choo et al. 2017 [18]	The authors performed a literature review of MMLs in the US.	The study was a literature review of MMLs and policy analyses.	None discussed.	The complexities and inconsistencies of MMLs, as well as the inherent characteristics of laws to undergo constant and rapid change makes them difficult to study.
Cohen et al. 2010 [28]	The authors performed a literature review of MC policies in the US.	Authors examined the various MC regulations in the US.	Physicians must act as gatekeepers of MC and should supervise the recommendation and distribution of MC. Further, physicians must be supervised by the board of medicine.	None discussed.
Davenport et al. 2016 [30]	Authors reviewed the implementation process, the Dangerous Drug Act (DDA) amendment. This was followed by a comparison of the statutory changes under DDA to other jurisdictions. Finally, other publicly available information and unstructured interviews with non-government stakeholders in Jamaica were also examined.	The authors drew on publicly available information and unstructured interviews with non-governmental stakeholders in Jamaica. Additionally, they cited publicly available government sources including secondary school and Jamaican household surveys. Information was also gathered from media reports including press releases and government statements.	The regulatory framework should also be revised to ensure that the demand for MC license reflects the number of applications, outlines the process and length of getting a license, highlights the demand for MC in the region, and the influence of MC regulations on tourism. The regulatory framework should also consider the influence of cultivators and retailers.	There must be clear guidelines for patients on which health conditions MC can be prescribed for, which health care practitioners are available for recommending MC, and what MC product is available for use.
Flexon et al. 2019 [19]	Authors conducted a multivariate regression analysis of data obtained from the National Survey on Drug Use and Health from 2015-2017 to determine whether medical marijuana laws had any effect on opioid use and misuse. A supplemental longitudinal panel analysis using data from the Interactive National Survey on Drug Use and Health State Estimates from 2002 to 2017 was also conducted.	The authors examined states that permit the medicinal use of cannabis.	In states that implemented MMLs, opioid reliance was effectively reduced.	None discussed.
Grbic et al. 2017 [20]	The study divided participants into two groups: group one consisted of government officials, lobbyists, medical professionals and group two consisted of researchers. Each group answered a questionnaire. Lastly, a third group consisting of members of the International Society for the Study of Drug Policy were interviewed. Data from each group were evaluated using thematic analysis.	The authors conducted a thematic analysis of the data extracted from the questionnaires and interviews with government officials, lobbyists, and medical professionals.	Authors found there to be a need for improved communication between researchers and policymakers to make evidence more accessible to the latter.	The lack of evidence within a political context and the lack of research on the actual implementation process of MC policies can act as a barrier.
Hammer et al. 2015 [21]	The study used data collected from medical marijuana websites, phone conversations, and public election and census results to develop a framework testing political culture as a proxy for social construction and the relationship between public attitudes and the implementation of medical marijuana laws.	The authors used data collected from medical marijuana program websites and phone conversation and survey results, as well as, data from 2010 Census Estimates and 2008 county election returns. Data obtained was regarding the MC program structure and implementation factors.	The implementation of MC regulations must take into consideration the role of local cultural factors during decision-making. The target population for the MC regulations must be seen as socially constructed groups. Finally, the MC implementation process includes patients advocates, the public health community, and law enforcement.	None discussed.
Heddleston et al. 2013 [15]	The study used data extracted from interviews, archival research, meeting observations, and official city documents to explore the varying experiences of San Francisco, Los Angeles, and San Diego regarding official responses to medical marijuana providers. The author also used such information to determine how the ways that activists open political opportunity structures contribute to regulatory approaches to medical marijuana.	Data for the case studies were collected from interviews with individuals who played a vital role in the MC regulation implementation process. The data also included information from archival data, literature reviews, and observational notes from the city council Cannabis Task Force meetings.	MC regulations are facilitated by ensuring law enforcement understands and sympathizes with the movement, establishing regulatory committees that can ensure the regulation of MC, creating local ballot initiatives, and having a city council that is pro-MC. According to the San Francisco Bay case study, the use of rallies and participation in city task forces further facilitated MC regulation. The model also showed that lobbying could aid with the facilitation.	According to the Los Angeles model, the absence of local ballot initiatives and inconsistent city council regulations, dispensaries became commercialized. As a result, social movements were not able to lobby for revised MC regulations. The San Diego model highlighted that the lack of local ballot initiatives, absence of MC regulations, and unsympathetic local law enforcement and city officials made it difficult to establish an MC regulatory framework.
Kim et al. 2018 [13]	Authors conducted a policy analysis through a mixed methods approach through qualitative data extracted from news reports searched by Los Angeles Times Archives, Chicago Tribune Archives, Denver Post News Archives, NewsBank and Google News Archive Search and quantitative information obtained from state level annual data.	The authors used the extracted data to undergo a thematic analysis and examine the three policy models: morality, economic, and multidimensional policy models.	States that utilized a ballot initiative tool, including California, Alaska, Oregon, Washington, Maine, and Colorado, found it to facilitate the MC law implementation process. Moreover, states that aim to stimulate economic goals will implement an MC law while states that have the support of cannabis users also increases chances of implementation. It was noted that according to the morality model, states with higher uses of cannabis and liberal-minded citizens have a higher likelihood of adopting an MC law. According to the economic policy model, states that have faced low fiscal capacity growth, have high incarceration rates, or have high costs associated with their justice system, will be more likely to implement a MC law. Similarly, states without a mandatory minimum sentencing law and smaller regulatory bureaucracy also show an increased likelihood to implement an MC law.	None discussed.
Lamonica et al. 2016 [9]	The authors used data extracted from analysis of ethnographic fieldwork that included observation notes from public meetings and in-depth interviews with stakeholders. Finally, data was also collected from interviews with stakeholders who followed policy development closely.	The authors used data from the MC policy implemented and regulated by the Massachusetts Department of Public Health.	The implementation of MC regulations can be facilitated through understanding the needs of stakeholders. Policymakers must ensure transparency and clear communication during the process; communication through ballot initiatives is an effective way of relaying information between politicians/policymakers and stakeholders and patients. The regulation must also consider and include MC education to ensure that it is not misunderstood by those interpreting it.	The lack of transparency and ineffective communication and education regarding MC regulations can lead to a misunderstanding of the information provided within them.
Lucas et al. 2008 [1]	The authors conducted a policy analysis using an evidence-based review of three facets of Health Canada's medicinal cannabis policy and the federal cannabis production and distribution program, in addition to examining Canada's network of unregulated community-based dispensaries.	Data for the study was collected from Canada's court decisions, government records, relevant studies, and network of unregulated community-based dispensaries. Moreover, the authors reviewed the Access to Information Act and the following policies: the Marihuana Medical Access Division (MMAD), the Canadians Institute of Health Research Medical Marijuana Research Program, and the federal cannabis production and distribution program.	The government must work with community-based medical cannabis compassion clubs, address safe and effective access to MC, and increase clinical research to address patient concerns.	None discussed.
Lucas et al. 2012 [22]	Authors used patient surveys and semi-guided interviews to assess the patient experience associated with Health Canada's MMAD. The data was then analyzed to determine the experiences and associated challenges with the program.	Data on Health Canada's MMAD and the quality of the service provided by the program was collected from a fifty question online survey along with twenty participants given semi-guided interviews.	The challenges faced by Health Canada's MC program can be ameliorated by increasing patient engagement and involvement, redirecting the responsibilities of MC towards healthcare professionals creating a community-based model by collaborating with local dispensaries, and increasing research on MC and its effects.	Challenges to patient access to MC include the absent role of the healthcare/medical community as a gatekeeper to MC, the burdensome application process and legal threats and issues caused by the federal government regarding MC.
Miyaji et al. 2016 [23]	The authors conducted a literature review of the archived official documents after World War II (1945–1948). This was followed by an analysis of the events that led to the implementation of MC regulations.	Authors extracted data from nationally archived official documents associated with the Cannabis Control Act (CCA). The documents were first developed at the end of World War II (1945 to 1948).	The development of an MC regulatory framework can be facilitated by reforming Article 4 of Cannabis Control Act (CCA). Regulations should ensure research opportunities to reduce any resistance in drug development, and create compassionate use programs.	None discussed.
Pacula et al. 2002 [31]	The authors conducted original legal research on the current state MC laws. The analysis was followed by an analysis of the fifty states and their MC regulations by comparing them with other dimensions.	Authors collected evidence from the Controlled Substance Act and Marijuana Policy Project.	None discussed.	States must ensure that a MC law that regulated MC supply must not increase recreational cannabis. Regulatory bodies must also take into consideration the medical necessity defense in state courts when implementing MC laws.
Pacula et al. 2014 [14]	The authors used public versions of the laws and examined the information in the laws using a systematic content analysis approach. The focus of the analysis was on determining when different factors of the laws were established, followed by an analysis of how the laws impacted access.	The authors analyzed all MMLs of 50 states enacted from 1990 to 2012.	In order to create regulations that are effective and efficient and to understand the outcomes of such mechanisms, there must be more empirical research on how patients respond to MMLs.	Policymakers find it challenging to establish an MC regulation program due to the illegal status of MC at the federal level that prevents MC from being treated as a medical product and regulated by the Federal Drug Administration. The inconsistencies in MMLs between states pose challenges for public health.
Pardo et al. 2014 [24]	Authors collected data from recent laws and regulations, discussions with the regulators in Uruguay, and the US states of Colorado and Washington. The data was then analyzed and compared in terms of cannabis prices, taxation, and supply and production.	The authors examined laws, regulations, and discussions with regulators and functionaries of each jurisdiction.	MC regulatory frameworks must reflect MC reforms and their influence on price and tax structures on MC regulation.	MC regulatory frameworks are challenged by the lack of evidence on the impact MC reforms may have. The taxation of cannabis can impact sales by making the product expensive for consumers. In Uruguay, it was found that a low market price for cannabis hindered revenue generation and may not influence the removal of illegal cannabis markets.
Smith et al. 2013 [17]	The authors used a case study method to analyze the Medical Marijuana Act (MMA) and its legitimacy through face-to-face and phone call interviews with the participants discussed previously.	The authors used face-to-face interviews with experts in the medical marijuana law field and attorneys and advocates involved in MC issues, as well as an analysis of court cases regarding the MMA.	It is important to ensure safe access to MC and protect the rights and privacy of patients and caregivers.	Due to the inconsistency and contradictions between the Schedule 1 classification of cannabis at the federal level and the MC laws in Michigan, the legitimacy and administration of MC at the state level is challenged. It is difficult to interpret and regulate MC laws because of this ambiguity.
Taylor et al. 2016 [25]	The authors collected previously unknown archival data to analyze changing attitudes towards the control of cannabis, the relationship between science and policy, and the impact of the policy environment on the process of re-medicalization.	The authors collected archival data from the Advisory Council on the Misuse of Drugs held at the National Archives from 1972–1982.	Re-medicalization of cannabis can be accomplished by increasing the amount of research conducted on MC, ensuring a relaxed stance towards the drug, and removing MC from drug control.	None discussed.
Tilburg et al. 2019 [16]	The authors analyzed the impact of federal restrictions on various aspects of regulation development in the cannabis industry.	Authors analyzed and collected evidence from state vs. federal MML policies.	None discussed.	Due to the conflict between the categorization of MC as a Schedule 1 drug in the USA and the legalization of cannabis at the state-level makes regulating MC difficult. Moreover, due to the lack of federal involvement in the cannabis industry, the development of a regulatory framework for MC research is negatively impacted.
Zarhin et al. 2018 [29]	Authors used interviews with stakeholders in the MC policy field, policy documents, and conference observations to highlight the dynamics between rhetorical and regulatory boundary-work. Data was also extracted from government resolutions on MC, information from Form 106, and data from an information booklet titled “Cannabis for medicinal use: An information booklet and medical guidelines”.	The authors drew information from the interviews with key stakeholders in the MC policy field, formal policy documents including Form 106, and observations of MC conferences.	Authors found that having an MC license system authorized by the state, the use of expert knowledge or the use of physicians as gatekeepers acts as a facilitator legitimizing MC.	None discussed.

### Finding from thematic analysis

The data extracted from this search was organized into the following themes, which included: (1) the effects of conflicts, mindset, and ideology of the state population, (2) the use of comparisons to analyze MC regulations, and (3) the need for more knowledge, advice, and empirical evidence to inform future MC policies.

#### Effects of conflicts, mindset, and ideology of the state population

Upon reviewing the 22 eligible articles, we found that 10 discussed the impact of conflict and/or population mindset on the implementation of MC regulation. Citizen-specific characteristics such as liberal-belief systems [3, 13] and higher cannabis use [13] were found to encourage MC law instatement while widespread faith in Christianity was a barrier [14]. State-specific factors such as neighbouring MC laws could act as facilitators or barriers as the adoption of a policy was more likely if it existed in a neighboring state [3], while ballot initiative measures often facilitated MC laws. More specifically, ballot measures provided opportunities for stakeholders, politicians, and policymakers to address the needs of citizens in their political work, acting as a facilitator for MC regulation [9, 13, 15]. We also found conflicts between state and federal scheduling of MC regulation to be a barrier and a source of regulation heterogeneity. Incongruence between federal and state governments made it more difficult to create a MC regulation system [14, 16], questioned administrative legitimacy and threatened MC laws [17], and infringed upon MC clinical research [15, 16, 18]. A unique conclusion drawn from one study was that MML implementation reduced opioid overreliance, potentially prompting states to view future MC regulation through a positive lense [19]. In demonstrating that state mindset can act as a barrier or facilitator, it is evident that successful MC regulation calls upon greater transparency, a better understanding of stakeholder concerns, and a more effective method of communicating regulation and political implications [9, 20].

#### Use of comparisons to analyze MC regulation

We found that a commonly used method for evaluating MC regulation was through the use of comparisons. More specifically, authors conveyed research comparing cannabis dispensaries, different US state MC laws, and individual country MC laws.

Cannabis dispensaries were used to study MC policy development through in-depth interviews with MC dispensary entrepreneurs [9], critical evaluations of unregulated community dispensaries [1], and structural examinations of dispensaries [21]. Cannabis dispensaries were also frequently cited as potential facilitators if successfully incorporated into community-based models of MC regulation or compassionate use programs [1, 22, 23].

Researchers reviewed how the adoption of MMLs spread across US states affected policy diffusion [3, 13]. A comparison across states in multiple articles reiterated the influence of neighboring states in the adoption process [3, 13]. Individual states faced barriers in creating an MC regulation system as it requires federal approval [14]. Comparisons between countries allowed for MC regulation to be conceptualized in different jurisdictions. This contributed to an understanding of barriers to MC policy implementation including the need to consider commercialization and empirical evidence on the effects of MC reforms [24]. Analyses comparing MC laws in major cities and multiple states were conducted by multiple researchers to gain a better understanding of the array of differences in MC regulation that can exist between jurisdictions [3, 13–15, 24].

#### The need for more knowledge, advice, and empirical and clinical evidence to inform future MC policies

Our analysis showed that many authors called upon the development of future MC policies with a greater degree of supporting evidence through varying means, including: the implementation of empirical and clinical evidence, medical community and physician suggestions, advice from policy officials and experts, and changes to improve patient experiences.

In an effort to include empirical evidence in future MC policies, further research regarding effects of MC reforms proposed included price and tax structures [24], increased cannabis research for re-medicalization [25], and bridging the lack of current political evidence were suggested [20]. Three studies mentioned the reciprocal impacts of MC regulation and clinical research. The classification of MC under the *Controlled Substance Act* in the US prevents the MC clinical research required for further legislative actions such as MC reclassification [26]. Increasing MC clinical research to better understand therapeutic mechanisms and ways to combat drug development lapses could encourage innovation and contribute to MC policy formation [23, 27].

The integration of the medical community into policy decision-making processes and establishing physicians as gatekeepers was proposed by multiple studies [22, 27–29]. Due to their medical expertise, prescribing authority, and ability to monitor other FDA-approved drugs [28], it has been recommended that physicians be designated as MC gatekeepers, replacing state governments in this position [29]. It was suggested that physicians’ reluctance to  involve themselves with MC leads to decreased safe patient access to MC [22], while their willingness to engage could prevent the spread of misinformation surrounding MC [28] and facilitate its safe and effective use among patients [29]. Accordingly, neglecting the input of these healthcare providers could have negative consequences and should be considered in future MC policies.

A common theme noted between five studies was the implied value of the advice and expertise of policy officials and relevant stakeholders in order to facilitate MC regulation. Strategic planning to establish legitimacy and indications for cannabis use may be facilitated through a multidisciplinary expert committee [25], healthcare professionals [29], medical associations [27], law enforcement [15, 21], and patient advocates [21, 29].

We found six studies which emphasized revising MC policy implementation processes to improve patient and community experiences. For example, prioritizing patient-centered approaches to care may improve challenges faced by Canadian MC programs [1], while patient protection from prosecution and arrest also has the potential to establish a functional Michigan MC program [17]. In this context, an MC program can be defined as a system for access to MC through a centralized, government-administered plan [1]. Further, a deeper understanding of the effects of governmental institutions on target populations such as retailers and cultivators [21, 30], and the incorporation of patient advocates in the implementation of MC policies [20] are both ways in which MC policies may support the community at-large. In order to facilitate better patient experiences, however, there is a need to address gray areas surrounding the clinical utility of MC for managing diseases and symptoms [27]. In addition, evidence suggests ambiguous MC laws that do not specify a source for MC may implicitly encourage patients to obtain MC through illegal channels [31]. As such, effective MC laws should provide information about MC sources to reduce the risk of punishment and prioritize patient safety [31].

## Discussion

### Significance of findings and comparative literature

The purpose of the present scoping review is to identify facilitators and barriers to MC regulation. Among the facilitators and barriers we identified, a body of comparative literature exists which supports the present review's findings. One such facilitator includes a given population viewing MC itself in a positive light. Authors of a qualitative study reported that positive public attitudes about MC in Israel and Norway were a particularly important factor which supported MC legalization [2]. In addition, evidence demonstrates that countries that allow MC usage under certain conditions have higher rates of public support for its legalization in comparison to countries that completely forbid the use of MC, identifying a possible reciprocal relationship [32]. A recurring barrier we identified included the need for more clinical and political evidence to inform future MC policies; the National Academies of Sciences, Engineering, and Medicine outlined that further evidence is required for US policymakers to make sound decisions regarding the use of cannabis in their 2017 national report [33]. In addition, Fitzcharles et al. emphasized that there is a need for more sound clinical evidence on the benefits and risks of MC to inform the advice of physicians and the work of policy regulators [34]. Additionally, researchers reported that individuals in favour of MC supported its legalization as they felt it would become easier to study and allow for a thorough investigation into its therapeutic benefits [32]. This complements the findings within our paper which suggest that MC policies require support from a greater degree of empirical evidence and clinical research in order to facilitate MC regulation.

The majority of our included articles (16 of 22) discuss MC regulations within the US, thus the themes which emerged from the present review may not be as generalizable to other jurisdictions. Our findings indicate that there is a large base of research focused on the US as the majority of our included articles (16 of 22) discuss MC regulations within the US. There exists several jurisdictions in which MC is legalized, however, we did not find any literature evaluating their MC regulations in a manner consistent with our eligibility criteria. For example, countries including, but not limited to, Austria, Belgium, Croatia, Denmark, and Spain, which have authorized MC use [35], however, we identified no literature evaluating their MC regulations. As such, it is necessary for future research to investigate MC regulatory frameworks in other countries in order to obtain a better understanding of facilitators and barriers on a global scale.

### Strengths and limitations

A notable strength of this scoping review included the use of a systematic search strategy to identify a comprehensive pool of synthesized evidence. The interpretation of these findings was strengthened by the use of two assessors who independently partook in the following steps: title/abstract screening, data extraction, and summarization of findings. A limitation of this review includes the fact that we excluded non-English literature which may have resulted in the omission of pertinent research conducted on this topic from countries where English is not a national language/widely-spoken. We did not search the gray literature given that we chose to assess the contents of articles which evaluated MC regulations which are typically found in the peer-reviewed literature.

## Conclusion

The present scoping review involved a systematic search of the literature to identify facilitators and barriers to MC regulation. We provide a comprehensive overview of various factors that influence the MC regulation process while highlighting a number of important themes including: (1) the effects of conflicts, mindset, and ideology of the state population, (2) the use of comparisons to analyze MC regulation, and (3) the need for more knowledge, advice, and empirical/clinical evidence to inform future MC policies. Policymakers should be aware of the facilitators to the MC regulation implementation process, such as the influence of state and federal congruence, increased transparency, and consideration of stakeholder concerns, in order to effectively respond to a growing societal acceptance of MC and its use among patients. In doing so, these efforts have the potential to overcome barriers to the MC regulation implementation process, including the influence of religiosity and a lack of communication between researchers and policymakers. Through a comprehensive understanding of these influential factors, policymakers will be better equipped to meet the consumer and commercial demands of a rapidly evolving MC regulatory environment.

## Data Availability

All relevant data are included in this manuscript.

## Abbreviations

MC: Medical cannabis
MML: Medical marijuana laws
